# *RPS24* microexon isoform as a novel biomarker for estrogen receptor-positive breast cancer progression and therapeutic resistance

**DOI:** 10.1038/s12276-025-01578-y

**Published:** 2025-11-15

**Authors:** Jiyeon Park, Dahye Nam, Seung-Hyun Jung, Bin Tian, Yeun-Jun Chung

**Affiliations:** 1 Precision Medicine Research Center, College of Medicine, The Catholic University of Korea, Seoul, Republic of Korea; 2 Department of Microbiology, College of Medicine, The Catholic University of Korea, Seoul, Republic of Korea; 3 Department of Biochemistry, College of Medicine, The Catholic University of Korea, Seoul, Republic of Korea; 4 Integrated Research Center for Genomic Polymorphism, College of Medicine, The Catholic University of Korea, Seoul, Republic of Korea; 5 Genome Regulation and Cell Signaling Program, The Wistar Institute, Philadelphia, PA USA

**Keywords:** Cancer genomics, Predictive markers

## Abstract

Alternative splicing significantly contributes to gene expression heterogeneity and disease progression, yet analyzing its dynamics in short genetic regions such as microexons remains challenging. Here we identified notable variations in ribosomal protein S24 (*RPS24*) splicing patterns across breast cancer subtypes and investigated this novel regulatory mechanism. To overcome the complexity of analyzing three consecutive microexons (3 bp, 18 bp and 22 bp), we developed a specialized approach combining splice junction read analysis with fragment analysis for accurate isoform quantification. We observed distinct isoform compositions across breast cancer cell lines. The 3-bp exon-containing isoform (ex4:3 bp) of *RPS24* showed significantly higher expression in estrogen receptor-positive (ER^+^) cells, demonstrating the strongest association with estrogen receptor signaling among all analyzed genes. This isoform functions as a molecular sensor for therapeutic interventions, being consistently upregulated following mTOR or CDK4/6 inhibition but consistently reduced across diverse drug-resistant cell lines, regardless of resistance mechanism. Through systematic RNA-binding protein screening and crosslinking immunoprecipitation followed by high-throughput sequencing analysis, we identified PTBP1 as a critical upstream regulator mediating microexon skipping. Analysis of multiple patient cohorts demonstrated that decreased ex4:3 bp expression strongly correlated with poor epithelial differentiation and metastatic progression specifically in ER^+^ breast cancer. Our findings suggest that *RPS24* alternative splicing is associated with a multilayered regulatory network integrating ER signaling, cell cycle and PTBP1-mediated splicing. The ex4:3 bp isoform serves as a potential biomarker for drug resistance and treatment response in ER^+^ breast cancer.

## Microexon analysis reveals role of RPS24 in drug resistance

This study explores how a gene called *RPS24*behaves differently in breast cancer. The authors wanted to understand how changes in *RPS24* might affect cancer progression and treatment response. The team analyzed data from breast cancer cell lines and patient samples to study the different forms of *RPS24*. They used RNA sequencing and experimental techniques to identify these isoforms. The researchers found that one specific isoform, called ex4:3 bp, is linked to estrogen receptor status in breast cancer. This means it might play a role in how breast cancer cells respond to hormone therapies. They also discovered that a protein called PTBP1 helps to regulate these isoforms by controlling which parts of the gene are included or skipped. This study suggests that the ex4:3 bp isoform could be a useful marker for predicting how breast cancer will respond to treatment.

This summary was initially drafted using artificial intelligence, then revised and fact-checked by the author.

## Introduction

Alternative splicing (AS) allows a single gene to produce multiple mRNA isoforms, contributing considerably to gene expression heterogeneity and phenotypic diversity^1^. This mechanism plays crucial roles in development, differentiation and disease progression. Despite the importance of AS in gene regulation, understanding AS dynamics in large-scale transcriptome data remains challenging. While many tools support AS analysis in RNA-sequencing (RNA-seq) data, their results often differ owing to varying algorithms and models^2,3^. Analysis accuracy further decreases when splicing regulation is complex or when the genetic regions regulated by AS are too short.

We previously developed a database called AS-CMC, designed to facilitate exploration of AS changes in cancer subtypes recorded in The Cancer Genome Atlas (TCGA)^4^. Within this database, subtype-specific AS patterns were commonly found in the ribosomal protein S24 (*RPS24*) gene in 13 out of 24 cancer types, suggesting its potential role in cancer-specific regulation. The *RPS24* gene has three microexons, each less than 30 nucleotides in length. The short length of these microexons renders their detection difficult as they can be missed or misaligned during read mapping. In addition, the combination of multiple short exons makes it challenging to identify and quantify all possible AS events. Therefore, many studies have focused on the inclusion and skipping of the 22 bp exon in the *RPS24* gene, which is the longest among the three^5–7^. Olivieri et al. conducted the first comprehensive analysis of all three microexons in the *RPS24* gene across 12 tissues using single-cell analysis, revealing its remarkable tissue specificity^8^. Our group has further analyzed *RPS24* AS isoform composition across cancer types and reported its relevance in epithelial–mesenchymal transition and response to KRAS-targeted therapy in cancer^9,10^.

Breast cancer is a highly heterogeneous disease with varied molecular and clinical characteristics^11,12^. Eukaryotic ribosomes, once perceived as uniform cellular machinery for protein synthesis, are now recognized for their regulatory roles in gene expression. As the roles of ribosomal proteins in breast cancer progression and treatment response continue to emerge^13,14^, understanding *RPS24* AS behavior in breast cancer could provide valuable insights into disease mechanisms.

Here, we aimed to examine *RPS24* AS patterns across breast cancer cell lines and associated isoforms, components and regulatory mechanisms in estrogen receptor (ER)-positive (ER^+^) breast cancer. The objective was to investigate the clinical significance of *RPS24* AS patterns by exploring its potential as a predictive biomarker for drug resistance and epithelial differentiation status in ER^+^ breast cancer.

## Materials and methods

### Data source for cell lines

Raw RNA-seq data in fastq format from the Cancer Cell Line Encyclopedia (CCLE) were downloaded from the Sequence Read Archive (SRA) under project PRJNA523380 (https://www.ncbi.nlm.nih.gov/sra). Cell line annotation, gene dependency and other processed genomic data were obtained using the depmap R package from Bioconductor (https://bioconductor.org). For RNA-seq datasets from non-CCLE cell lines, fastq files were acquired from SRA through searches on GEO (https://www.ncbi.nlm.nih.gov/geo) or ArrayExpress (https://www.ebi.ac.uk/biostudies/arrayexpress) websites. Initial data in SRA format were converted to fastq files using the SRA Toolkit (https://www.ncbi.nlm.nih.gov/home/tools).

### Sources for patient data

For the cohort analysis of patients with breast cancer, we obtained transcriptome profiling data from the TCGA (*n* = 1,098 patients)^15^, Clinical Proteomic Tumor Analysis Consortium (CPTAC; *n* = 134 patients)^16^ and Metastatic Breast Cancer (MBC; *n* = 200 patients) project^17^ through the Genomic Data Commons (GDC) portal (https://portal.gdc.cancer.gov). We downloaded splice junction quantification files generated by the GDC bioinformatics pipeline after dbGaP approval. Additional TCGA sample data were accessed using the TCGAbiolinks R package in Bioconductor^18^, and CPTAC sample data were obtained using the CPTAC Python package (https://paynelab.github.io/cptac). For the MBC project, corresponding clinical data were acquired from cBioPortal (https://www.cbioportal.org).

### *RPS24* AS analysis using splicing junction reads

The fastq files were mapped to the reference genome (hg38) using the STAR aligner in two-pass mode^19^. We extracted information from the SJ.out.tab files, which summarize splice junctions and serve as output files from the STAR alignment process. *RPS24* AS isoform analysis was performed following the protocol outlined in a recently published paper^10^. For each sample, we extracted splice junction reads at the 5′ splice site (ss) of exon 4 and categorized them on the basis of the 3′ ss. As exon 4 is constitutive, we considered the total number of reads with the exon 4 5′ ss as *RPS24* gene expression and reads corresponding to the 3′ ss as expression of each isoform. To assess isoform proportions, we calculated the ratio of junctional reads representing each isoform to the total number of junctional reads starting from exon 4.

### Crosslinking immunoprecipitation followed by high-throughput sequencing data analysis

We downloaded raw fastq files from a crosslinking immunoprecipitation followed by high-throughput sequencing (CLIP-seq) dataset (GSE220184). The sequencing reads were aligned to the human reference genome (hg38) using the Burrows–Wheeler Aligner (version 0.7.17) with default parameters. The resulting SAM files were converted to BAM format, sorted by genomic coordinates and indexed using Samtools (version 1.13). To generate genome coverage files suitable for visualization, we processed the sorted BAM files using bamCoverage from the deepTools package (version 3.5.5). The generated bigWig (bw) files were loaded into the Integrative Genomics Viewer (version 2.19.1) for visualization and qualitative analysis. The visualization in Integrative Genomics Viewer allowed us to validate peaks on motifs identified by RBPmap (version 1.2), including a database of 274 RNA-binding motifs^20^. The PTBP1 binding sites were identified using the PTBP3 motif model in RBPmap, as PTBP1 and PTBP3 share highly similar RNA recognition motifs and sequence specificity^21^.

### Developmental lineage and stemness score analysis

Ectoderm, mesoderm, endoderm and stemness scores for TCGA breast cancer samples from patients were obtained using the TCGAanalyze_Stemness function from the TCGAbiolinks R package. The analysis workflow involved several steps: first, TCGA RNA-seq data were downloaded and prepared using the GDCquery and GDCdownload functions from TCGAbiolinks. The downloaded data were then normalized and filtered using TCGAanalyze_Normalization and TCGAanalyze_Filtering functions to remove low-expression genes and ensure data quality. Finally, stemness and lineage scores were calculated using specific stem cell signatures, including SC_PCBC_stemSig for stemness characteristics and lineage-specific signatures ECTO_PCBC_stemSig, MESO_PCBC_stemSig and ENDO_PCBC_stemSig for ectoderm, mesoderm and endoderm scores, respectively^22^. The stemness scores reflect the degree of oncogenic dedifferentiation, while the lineage scores indicate the developmental origin characteristics of tumor samples.

### Experimental validation of *RPS24* AS in breast cancer cell lines

We studied 11 breast cancer cell lines categorized by ER expression. ER^+^ lines included MCF7, T47D, HCC1428 and ZR751, whereas ER-negative (ER^−^) lines included HCC2218, HCC1954, MDA-MB-453, HCC1143, HCC1187, MDA-MB-231 and HCC38. Cells were cultured in Roswell Park Memorial Institute (RPMI)-1640 medium (HyClone) supplemented with 10% fetal bovine serum (HyClone) and 1% penicillin–streptomycin (Thermo Fisher Scientific). For mTOR inhibition studies, MCF7 cells were treated with 500 nM everolimus (Sigma-Aldrich) for 48 h. For drug resistance studies, tamoxifen-resistant MCF7 cells were obtained from the American Type Culture Collection (CRL-3435) and cultured according to the manufacturer’s instructions in DMEM medium (WELGENE) supplemented with 10% fetal bovine serum, 10 μg/ml of human insulin (Sigma-Aldrich) and 1 μM 4-hydroxytamoxifen (Sigma-Aldrich).

For fragment analysis of *RPS24* AS, cells were lysed using TRIzol (Thermo Fisher Scientific) followed by the TRIzol–chloroform extraction protocol. The AS region was amplified using previously published primers^23^, with the forward primer modified with FAM fluorescent dye for capillary gel electrophoresis detection. Primer sequences were as follows: *RPS24* ex4F:ATGGCCTGTATGAGAAGAAA and *RPS24* ex6R: CACAGCTAACATCATTGCAG. The fluorescently labeled PCR products were diluted to 1:100 and analyzed using an ABI 3130xl Genetic Analyzer (Thermo Fisher Scientific).

Through the Peak Scanner program (Thermo Fisher Scientific), fragment sizes were determined and peak areas were analyzed by measuring fluorescence intensity. Each peak represented an *RPS24* AS isoform, and the relative expression of *RPS24* AS isoforms was determined by calculating the ratio of peak areas.

## Results

### Characterizing *RPS24* AS isoforms in breast cancer cell lines

**Fig. 1:**
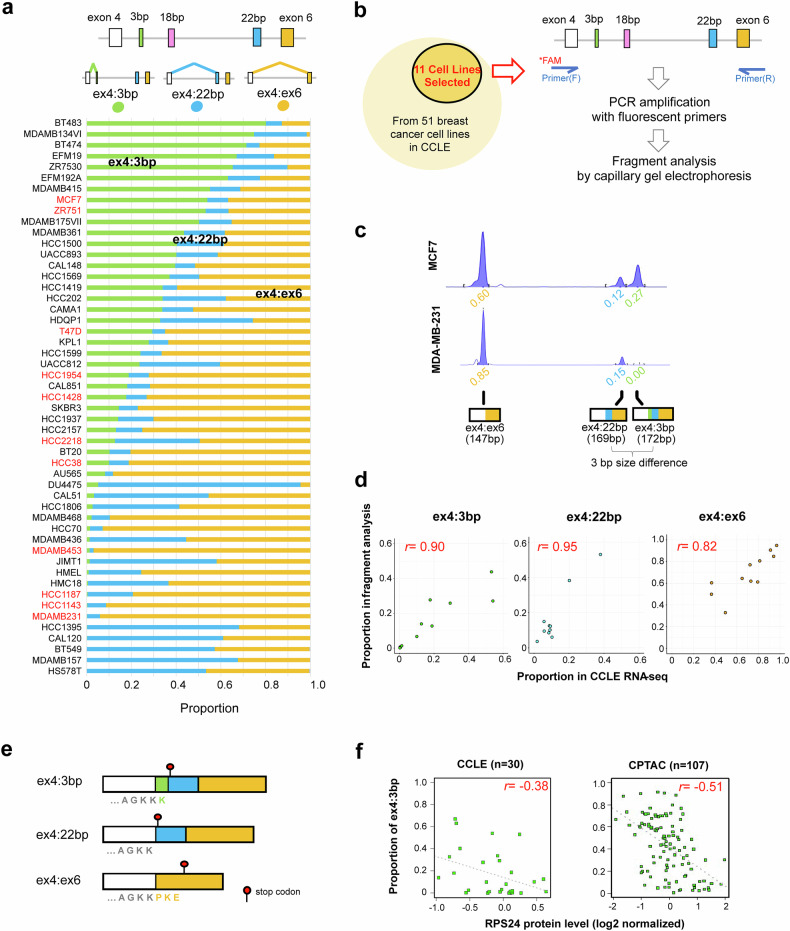
Analysis of *RPS24* AS in breast cancer cell lines. **a** Heterogeneity of *RPS24* AS isoform composition in 51 CCLE breast cancer cell lines. Top: the *RPS24* gene structure between exons 4 and 6. In RNA-seq data, junction reads derived from exon 4 (marked by colored lines) were used to determine the expression of each isoform. The stacked bar graphs below show the proportion of each isoform. Cell lines are ordered according to the ex4:3 bp isoform ratio. Cell lines highlighted in red were used for experimental validation (Fig.1d). **b** Fragment analysis by capillary gel electrophoresis for experimental validation. From 51 breast cancer cell lines in CCLE, 11 representative cell lines were selected for experimental validation. PCR amplification was performed with fluorescent primers, followed by fragment analysis by capillary gel electrophoresis to detect and quantify *RPS24* AS isoforms. **c** Representative electrograms for MCF7 and MDA-MB-231 cell lines are shown. The fragment types corresponding to each peak and their proportions are indicated below. Through fragment size analysis, we discovered that all transcripts containing the ex4:3 bp junction lacked the 18-bp exon but invariably included the 22-bp exon, with a 3-bp size difference between the two peaks. The area of each peak was quantified as the expression of each isoform, and the proportion of total expression accounted for by each isoform was calculated. **d** Correlation analysis between experimental results and CCLE RNA-seq data for the 11 cell lines. The *x*-axis represents values calculated from RNA-seq analysis, while the *y*-axis shows values obtained from fragment analysis. Scatter plots display the proportion of each *RPS24* AS isoform with corresponding Pearson correlation coefficients (*r* = 0.90 for ex4:3 bp, *r* = 0.95 for ex4:22 bp and *r* = 0.82 for ex4:ex6). **e** Protein sequences resulting from the three *RPS24* AS isoforms present in breast cancer. The ex4:3 bp and ex4:22 bp isoforms differ by just one lysine (K) residue, while the ex4:ex6 isoform generates a distinct C-terminal sequence with three additional amino acids (PKE). The red lollipop symbol indicates the stop codon position in each isoform. **f** Correlation between ex4:3 bp isoform proportion and RPS24 protein levels. Analysis of both CCLE breast cancer cell lines (*n* = 30, left) and CPTAC data from patients with breast cancer (*n* = 107, right) shows negative correlations (*r* = −0.38 and *r* = −0.51, respectively).

To assess the diversity among breast cancer cell lines, we first investigated the distribution of *RPS24* AS isoforms in 51 breast cancer cell lines from CCLE^24^ and observed profound heterogeneity in isoform composition between cell lines (Fig. 1a). Both the ex4:22 bp and ex4:ex6 junctions were present in 49 out of 51 cell lines (96% of total cell lines), with expressions exceeding 5%. However, the ex4:3 bp junction was detected in only two-thirds of the investigated cell lines (34 out of 51 cell lines). The ex4:18 bp junction was not detected, and when RNA-seq profiles were visualized, the 18-bp exon showed no expression (Supplementary Fig. 1).

To ensure the reliability of our *RPS24* AS isoform measurement, we performed two methods of validation. First, we compared the CCLE results with four independent RNA-seq datasets of breast cancer cell lines from GEO (GSE48213, GSE58135, GSE73526 and PRJEB30617) (Supplementary Fig. 2). For pairwise comparison, we utilized cell lines that overlapped between CCLE and the four datasets. The correlation was particularly strong for ex4:3 bp (median *r* = 0.92), whereas ex4:22 bp (median *r* = 0.81) and ex4:ex6 (median *r* = 0.81) also showed good correlation between CCLE and GEO.

Next, we experimentally determined the composition of *RPS24* microexons in 11 breast cancer cell lines (cell lines marked in red in Fig. 1a) using fragment analysis (Fig. 1b). In each cell line, we observed a maximum of three peaks, showing the types of isoforms generated by AS between exon 4 and exon 6. Through fragment size analysis, we discovered that all transcripts containing the ex4:3 bp junction lacked the 18-bp exon but invariably included the 22 bp exon. Therefore, the ex4:3 bp and ex4:22 bp junctions differ by only 3 bp, which the fragment analysis successfully separated and quantified (Fig. 1c). On the basis of these findings, we named the isoforms as follows: ex4:3 bp for the isoform containing both 3-bp and 22-bp exons, ex4:22 bp for the isoform containing only the 22-bp exon and ex4:ex6 for the isoform lacking all microexons. When comparing the proportion of each isoform calculated from RNA-seq data with experimental validation data from the same cell lines, both showed high concordance (*r* > 0.8) across all three isoforms (Fig. 1d). These results indicate that our bioinformatic method is a reliable approach for analyzing the composition of *RPS24* AS isoforms in RNA-seq data.

The three *RPS24* isoforms present in breast cancer exhibited variations in their C-terminal amino acid composition (Fig. 1e). The difference between ex4:3 bp and ex4:22 bp lies in the presence of one lysine (K), and translation terminates at the stop codon found in the 22-bp exon. By contrast, ex4:ex6 generates a protein ending with three amino acids: proline (P), lysine (K) and glutamic acid (E). To determine whether these differences in C-terminal amino acid composition affect protein abundance, we examined transcriptomics and proteomics data from CCLE breast cancer cell lines (*n* = 30) and CPTAC data from patients with breast cancer (*n* = 107). We found that transcripts containing the ex4:3 bp junction showed no correlation with *RPS24* RNA levels but exhibited a negative correlation with the protein levels in both datasets (Fig. 1f and Supplementary Fig. 3). These findings suggest that the ex4:3 bp isoform may undergo post-transcriptional regulation (for example, nonsense-mediated decay) or post-translational mechanisms (for example, altered protein stability).

### *RPS24* AS isoform composition is regulated by ER in breast cancer

**Fig. 2:**
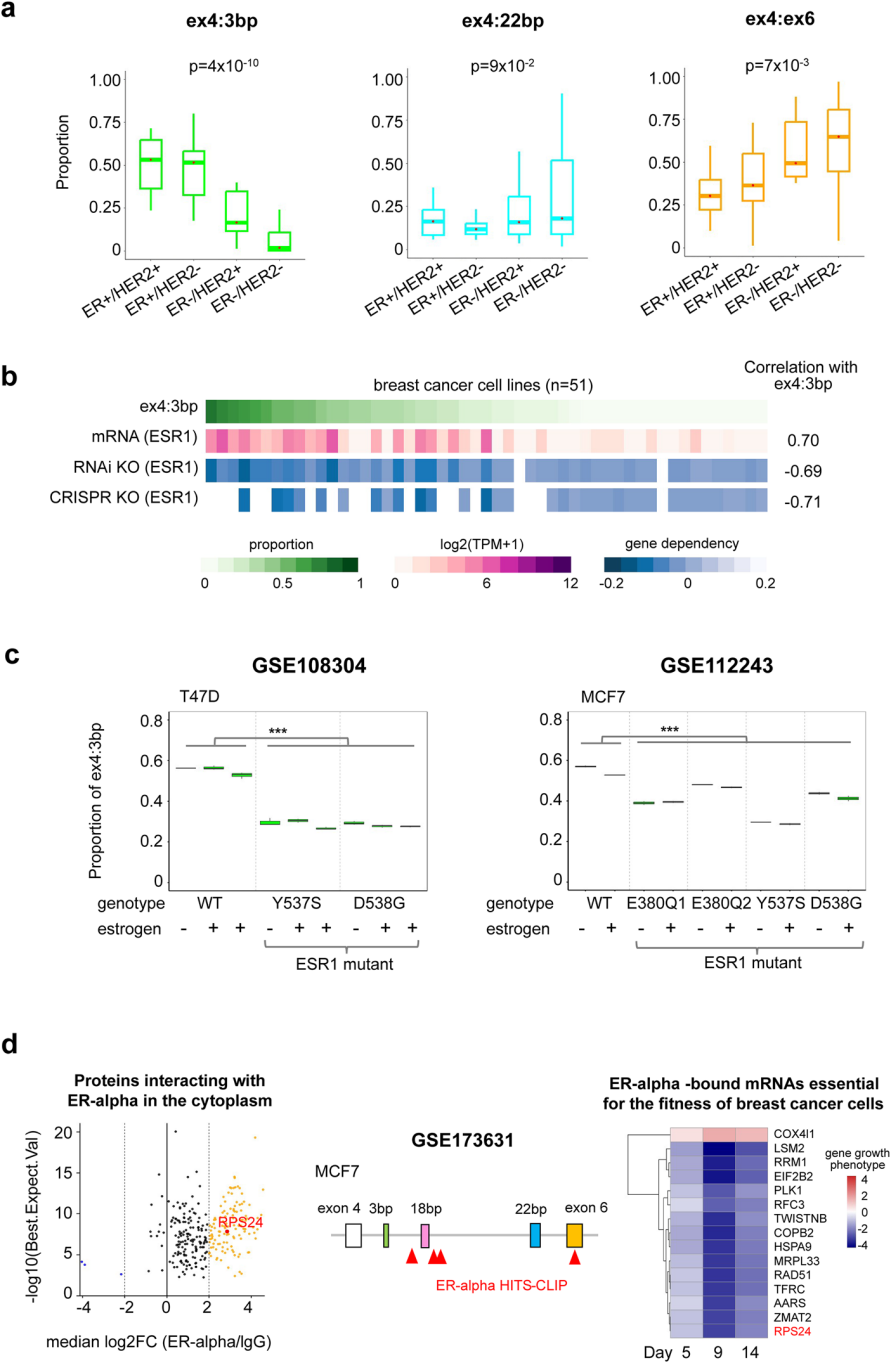
Association of *RPS24* ex4:3 bp isoforms with ER signaling. **a** Comparison of *RPS24* AS isoform expression between breast cancer cell line molecular subtypes. Cell lines are categorized into four types on the basis of ER and HER2 expression. The *y*-axis represents the proportion of each isoform. *P* values derived from analysis of variance tests are shown for each plot. **b** Association of the ex4:3 bp isoform with the *ESR1* gene. Columns sort cell lines by ex4:3 bp expression (green). Lower rows show *ESR1* mRNA expression and two gene dependency scores (KO by RNAi or CRISPR) in the corresponding cell lines. Correlation with ex4:3 bp is displayed on the right. **c** Changes in ex4:3 bp expression due to *ESR1* gene mutations. GSE108304: T47D cell line with Y537S and D538G mutations, treated with ethanol or 10 nM estrogen (estradiol, E2) for 4 h or 24 h. GSE112243: MCF7 with E380Q, Y537S and D538G mutations, with or without 24 h of estrogen (E2) treatment. Differences between *ESR1* wild type and mutants were analyzed using a *t*-test (^***^P < 0.0005, ^**^P < 0.005, ^*^P < 0.05). **d** ER-alpha interactions with *RPS24* at multiple levels (GSE173631, MCF7 cells). Left: proteins interacting with *ESR1* in the cytoplasm, identified by immunoprecipitation followed by mass spectrometry. The *x*-axis shows median log_2_ fold change (ER-alpha/immunoglobulin G (IgG)) in protein abundance between ER-alpha immunoprecipitation and IgG control, with higher values indicating stronger interactions with ER-alpha. The *y*-axis shows −log_10_(Best.Expect.Val), with higher values indicating greater statistical confidence of protein identification. Right: a heat map showing that *RPS24* (highlighted at bottom) is among *ESR1*-bound mRNAs essential for breast cancer cell fitness, as determined by CRISPR interference of *ESR1-*binding sites. The top 15 genes were selected on the basis of Mann–Whitney *P* value, and each cell value represents the average phenotype of the three strongest single-guide RNAs on cell growth across day 5, 9 and 14 time points compared with day 0. Negative values indicate stronger growth inhibition.

We investigated the differences in *RPS24* AS isoform expression across molecular subtypes in the CCLE breast cancer dataset. Analysis based on ER and human epidermal growth factor receptor 2 (HER2) status (ER^+^/HER2^+^, *n* = 6; ER^+^/HER2^−^, *n* = 12; ER^−^/HER2^+^, *n* = 8; ER^−^/HER2^−^, *n* = 24; unclassified, *n* = 1) revealed contrasting regulatory patterns between the ex4:3 bp and ex4:ex6 isoforms (*P* < 0.01), whereas the ex4:22 bp isoform showed minimal variation (*P* = 0.09) (Fig. 2a). Accordingly, further classification by molecular subtypes (luminal, *n* = 11; HER2_amp, *n* = 13; luminal_HER2_amp, *n* = 2; basal_A, *n* = 15; and basal_B, *n* = 9) demonstrated higher expression of the ex4:3 bp isoform in luminal subtypes and lower expression in basal subtypes (Supplementary Fig. 4). This finding suggests a potential association between ex4:3 bp expression and breast cancer subtypes, particularly relating to ER status.

To further elucidate the relationship between ex4:3 bp and ER expression, we examined the association between *ESR1* expression and its dependency on the *RPS24* ex4:3 bp isoform. Our analysis revealed a strong positive correlation between *ESR1* messenger RNA (mRNA) expression and ex4:3 bp expression (*r* = 0.7) (Fig. 2b). Furthermore, ex4:3 bp expression showed negative correlations with *ESR1* gene dependency scores, as determined by both RNA interference (RNAi) and CRISPR–Cas9 knockout (KO) methodologies (*r* < −0.6). These negative correlations indicate that cells expressing high levels of ex4:3 bp are more dependent on *ESR1* for survival. Notably, the correlation between ex4:3 bp and both *ESR1* mRNA expression and gene dependency scores ranked among the highest when compared with all other genes (Supplementary Fig. 5a), emphasizing the strong association between ex4:3 bp expression and *ESR1* gene function in breast cancer cells.

To further investigate the relationship between *ESR1* and *RPS24* AS, we analyzed transcriptome data from two luminal A-type breast cancer cell lines (MCF7 and T47D) harboring mutations in the ligand-binding domain of the *ESR1* gene, a common occurrence in metastatic breast cancer (Fig. 2c). Analysis of the GSE108304 dataset (T47D) revealed a significant decrease in the proportion of ex4:3 bp isoform in *ESR1* mutant cells (Y537S and D538G) compared with that in wild-type cells (*P* < 0.0005). Similarly, the GSE112243 dataset (MCF7) demonstrated consistent decreases in ex4:3 bp expression across various *ESR1* mutations (E380Q, Y537S and D538G); however, it depicted some variation depending on the mutation type (*P* < 0.0005). Estrogen treatment did not significantly alter the ex4:3 bp proportions within each genotype, suggesting that the effect of *ESR1* mutations on *RPS24* splicing occurs independently of estrogen stimulation (Fig. 2c). These findings were consistently observed across additional datasets, and we also noted a decrease in ex4:3 bp expression in *ESR1* fusion (Supplementary Fig. 5b–d), further confirming the relationship between ER signaling and *RPS24* splicing.

Our analysis of ESR1-related datasets (GSE173631) revealed multiple levels of ER-alpha interaction with *RPS24 (*ref. ^25^). ESR1 immunoprecipitation followed by quantitative mass spectrometry analysis demonstrated that ER-alpha physically interacts with *RPS24* protein in the cytoplasm (Fig. 2d, left). CLIP-seq data showed that ER-alpha directly binds to *RPS24* transcripts, specifically to the intronic regions between exon 4 and exon 6 and the 3′UTR (Fig. 2d, middle). When ER-alpha binding sites were repressed using CRISPR interference, *RPS24* emerged as one of the top 15 genes of which the loss significantly compromised breast cancer cell fitness (Fig. 2d, right). These data indicate that ER-alpha interacts with *RPS24* at both protein and RNA levels and that this interaction is critical for breast cancer cell viability.

### Differential regulation of ex4:3 bp isoform by cell cycle/mTOR inhibition and in endocrine therapy resistance

**Fig. 3:**
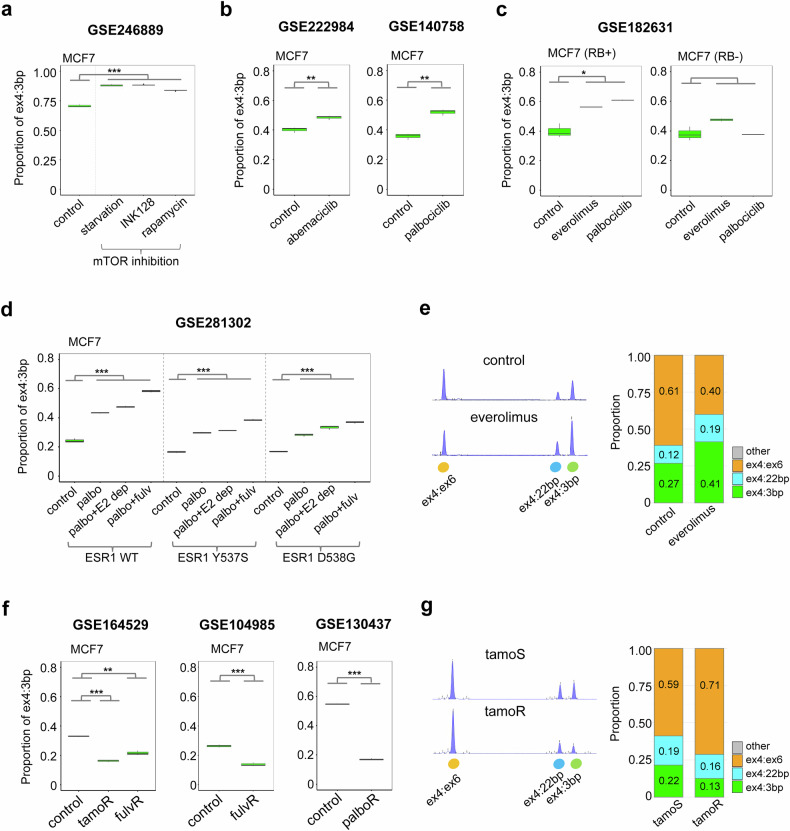
Regulation of *RPS24* ex4:3 bp isoform in response to pathway inhibition and drug resistance in breast cancer cells. **a** The effect of mTOR inhibition in MCF7 cells (GSE246889). The ex4:3 bp isoform proportion was compared among control and three mTOR inhibition conditions: starvation, INK128 and rapamycin. **b** The response to CDK4/6 inhibition. Left: MCF7 cells treated with abemaciclib (500 nM, 96 h) (GSE222984). Right: MCF7 cells treated with palbociclib (500 nM, 48 h) (GSE140758). **c** Comparison of the mTOR and CDK4/6 inhibition effects in RB-positive (left) and RB-negative (right) MCF7 cells (GSE182631). Cells were treated with everolimus (500 nM) or palbociclib (250 nM) for 48 h. **d** Effect of CDK4/6 inhibitor treatment conditions on ex4:3 bp isoform. MCF7 cells expressing wild-type *ESR1* (*ESR1* WT) or *ESR1* mutations (*ESR1* D538G and *ESR1* Y537S) were treated with vehicle control (DMSO), palbociclib (200 nM), palbociclib combined with estrogen-deprived media or palbociclib combined with fulvestrant (1 nM). Statistical significance was determined using a *t*-test. Gray brackets indicate statistical comparisons between control and palbociclib groups. **e** Fragment analysis validation of the mTOR inhibition effect. Left: representative electropherograms showing three *RPS24* isoforms in control and everolimus-treated (500 nM, 48 h) MCF7 cells. Right: quantification of isoform proportions. **f** Analysis of ex4:3 bp isoform in drug-resistant models. Comparison between parental and resistant cells for tamoxifen and fulvestrant resistance (GSE164529 and GSE104985) (tamoR and fulvR) and palbociclib resistance (palboR) (GSE130437). **g** Fragment analysis comparison between tamoxifen-sensitive (tamoS) and tamoxifen-resistant (tamoR) MCF7 cells. Left: representative electropherograms. Right: quantification of isoform proportions. ***P ≤ 0.0005, ** *P*≤ 0.005 and * *P* ≤ 0.05.

Having established the regulatory relationship between ER-alpha and *RPS24* AS, we next aimed to understand how this splicing event responds to therapeutic interventions and contributes to treatment resistance, which are critical aspects of breast cancer management. We investigated the regulation of the *RPS24* ex4:3 bp isoform in response to two key elements of breast cancer treatment: pathway inhibition and drug resistance. First, we examined the effects of inhibiting critical signaling pathways. In MCF7 cells, mTOR inhibition through INK128, rapamycin or starvation significantly increased the ex4:3 bp isoform (GSE246889, *P* < 0.0005) (Fig. 3a). Similarly, CDK4/6 inhibition using either abemaciclib (GSE222984) or palbociclib (GSE140758) led to increased ex4:3 bp isoform levels (*P* < 0.005) (Fig. 3b). These changes occurred only in retinoblastoma (RB)-positive cells (GSE182631, *P* < 0.05) (Fig. 3c), suggesting that the ex4:3 bp isoform may serve as an indicator of cell cycle pathway activity. The upregulation of ex4:3 bp following mTOR and CDK4/6 inhibition indicates that this isoform may be involved in cellular adaptation to growth-inhibitory conditions, potentially contributing to treatment response.

We further examined the effect of *ESR1* mutations on the ex4:3 bp isoform in response to CDK4/6 inhibition alone or in combination therapy. In MCF7 cells expressing wild-type *ESR1* (WT), D538G or Y537S mutations, we observed significant changes in ex4:3 bp isoform across all treatment conditions (GSE281302) (Fig. 3d). Treatment with palbociclib alone, palbociclib combined with estrogen deprivation (palbo+E2 dep) or palbociclib combined with fulvestrant (palbo+fulv) significantly increased the proportion of ex4:3 bp splicing compared with that in control (*P* < 0.001) in all *ESR1* variants. However, both *ESR1* mutants (D538G and Y537S) showed distinct splicing patterns compared with wild-type *ESR1*, suggesting that *ESR1* mutation status may influence the splicing response to CDK4/6 inhibition and combination treatments. To validate these findings, we performed fragment analysis in MCF7 cells, with results shown as the average of three independent experiments. These experiments demonstrated a 1.5-fold increase in the proportion of ex4:3 bp following mTOR inhibition with everolimus treatment compared with that in the control (Fig. 3e).

We then examined the behavior of the isoform in drug resistance models. We observed a consistent decrease in the isoform across various drug-resistant cell lines (Fig. 3f). This reduction was evident in cells resistant to different therapeutic agents: tamoxifen (GSE164529, *P* < 0.0005), fulvestrant (GSE164529 and GSE104985, *P* < 0.005) and palbociclib (GSE130437, *P* < 0.0005). These results suggest that downregulation of the *RPS24* ex4:3 bp isoform represents a common feature in the development of drug resistance in breast cancer cells, independent of the mechanism of action of the drug. We experimentally confirmed a 1.7-fold decrease in tamoxifen-resistant cells relative to tamoxifen-sensitive cells through fragment analysis (Fig. 3g).

### Identification of PTBP1 as an upstream regulator of *RPS24* AS

**Fig. 4:**
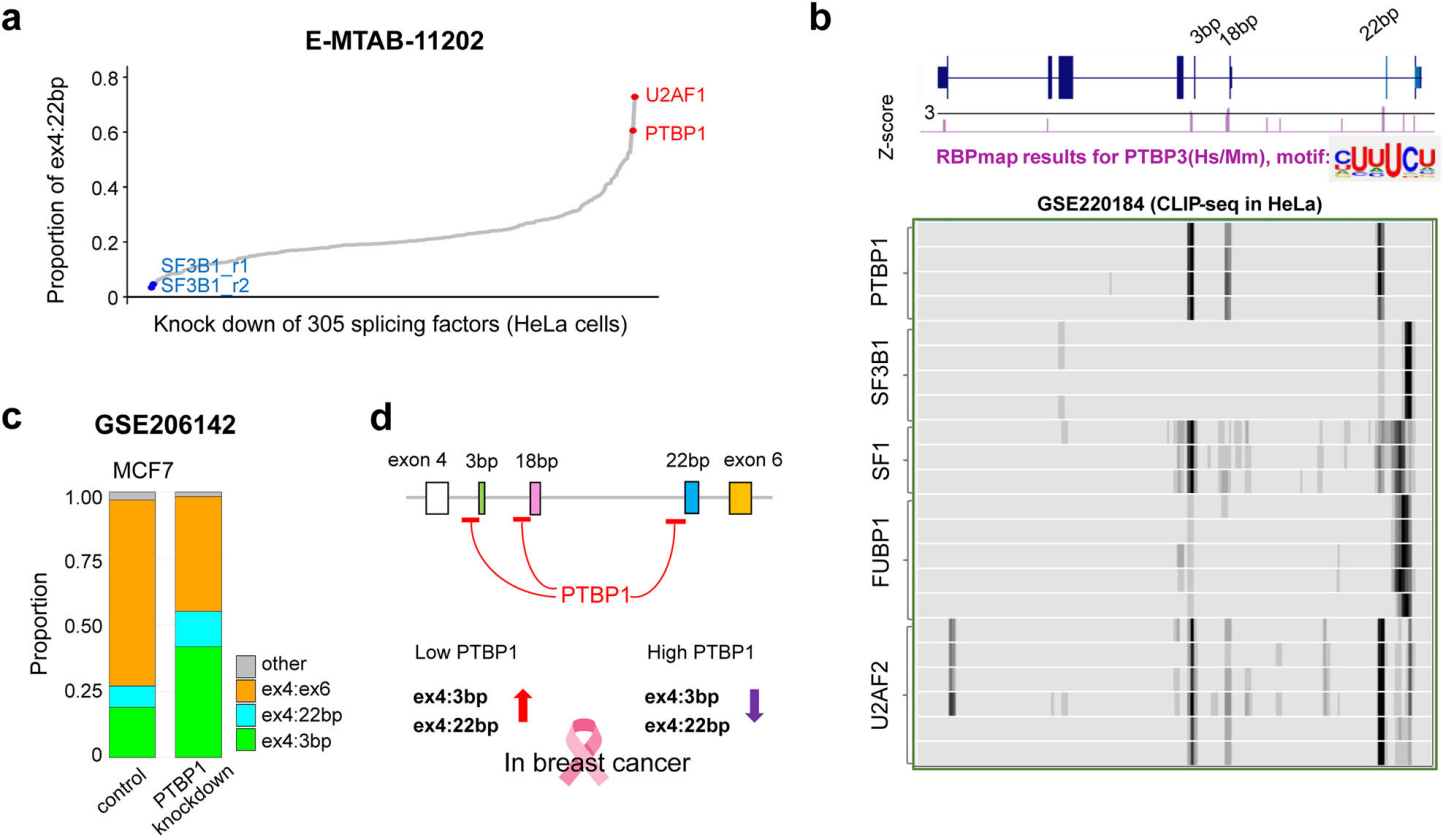
Identification of PTBP1 as a key regulator of *RPS24* AS. **a** The effect of systematic knockdown of 305 splicing factors on ex4:22 bp in HeLa cells (E-MTAB-11202). The *y*-axis shows the proportion of the ex4:22 bp isoform after each factor knockdown. *U2AF1* and *PTBP1* knockdown (red dots) markedly increased the ex4:22 bp proportion, while SF3B1 knockdown (blue dot) dramatically decreased it. **b** PTBP1 binding site analysis. Top: in silico prediction using RBPmap identifies multiple PTBP1 binding motifs (CUUUCU) in the polypyrimidine tracts upstream of all three microexons, with *z*-scores greater than 3 (red lines). Note: RBPmap uses the PTBP3 motif model, which is functionally equivalent to PTBP1 owing to shared binding specificity. High stringency (*P* value(significant) < 0.005 and *P*value(suboptimal) < 0.01) with a conservation filter was applied. Bottom: experimental validation using CLIP-seq data (GSE220184) from HeLa cells confirming PTBP1 binding at the predicted sites, with binding signals also observed for other splicing factors (SF3B1, SF1, FUBP1, U2AF1 and U2AF2). **c** The effect of *PTBP1* knockdown on *RPS24* AS isoform composition in MCF7 breast cancer cells (GSE206142). The stacked bar graph shows notable increases in both ex4:3 bp (2.2-fold) and ex4:22 bp (1.7-fold) isoforms following PTBP1 depletion. **d** A proposed model of PTBP1-mediated regulation of *RPS24* microexon splicing in breast cancer. PTBP1 binds to upstream intronic regions flanking the microexons and functions as a repressor of microexon inclusion. Under low PTBP1 conditions, microexon-containing isoforms (ex4:3 bp and ex4:22 bp) show increased expression, while high PTBP1 levels promote microexon skipping.

We sought to identify the splicing factors that regulate *RPS24* AS patterns. To this end, we leveraged a systematic splicing factor knockdown dataset (305 factors in HeLa cells)^26^. Since HeLa cells do not express the ex4:3 bp isoform, we focused on splicing factors that substantially altered the proportion of the ex4:22 bp isoform. The screening revealed that the knockdown of *U2AF1* and *PTBP1* led to a substantial increase in the proportion of ex4:22 bp, yielding proportions of 0.79 and 0.65, respectively, ranking as the top two most potent expression enhancers (Fig. 4a and Supplementary Table 1). By contrast, *SF3B1* knockdown replicate experiments (SF3B1_r1 and SF3B1_r2) showed the lowest proportion of this isoform (0.05 and 0.03, respectively), as shown by the blue dots.

In the RBPmap database, we identified PTBP1 binding motifs with *z*-scores greater than 3 in the polypyrimidine tract upstream of the 3′ ss of all three microexons, using a high stringency filter option (*P*value(significant) < 0.005 and *P*value(suboptimal) < 0.01) with conservation filter (Fig. 4b, top). To confirm these in silico predictions, we examined independent CLIP-seq data from HeLa cells (GSE220184). The CLIP-seq data showed clear enrichment of PTBP1 binding at all three microexon regions (particularly strong at the 3-bp and 22-bp positions), with robust signals at positions matching the predicted motifs (Fig. 4b, bottom). This observation was consistent across all replicates. Furthermore, the CLIP-seq data revealed that U2AF2 exhibited similar binding patterns.

To validate the role of these factors in breast cancer models, we observed notable increases in both ex4:3 bp and ex4:22 bp isoforms following PTBP1 depletion (Fig. 4c). We observed a 2.2-fold increase in ex4:3 bp expression (from approximately 0.2 to 0.45) and a 1.7-fold increase in ex4:22 bp expression (from approximately 0.1 to 0.17) in the MCF7 (GSE206142) cell line, with a corresponding decrease in the ex4:ex6 isoform. These findings suggest that PTBP1 acts as a repressor of microexon inclusion in *RPS24* (Fig. 4d) in breast cancer. In conditions with low PTBP1 protein expression, the microexon-containing isoforms appear to be derepressed, leading to their increased expression.

### Ex4:3 bp as a potential clinical biomarker in breast cancer

**Fig. 5:**
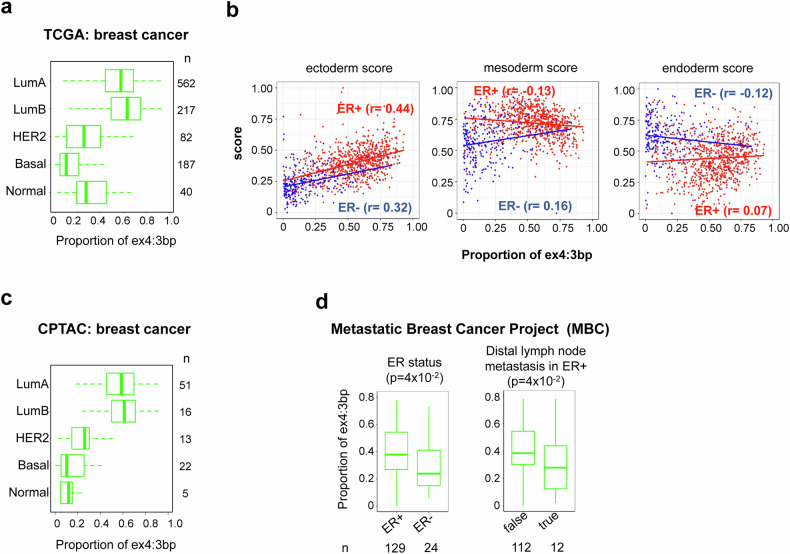
Clinical significance of the *RPS24* ex4:3 bp isoform in cohorts of patients with breast cancer. **a** The distribution of ex4:3 bp isoform proportions across molecular subtypes in TCGA breast cancer data, showing significantly higher expression in luminal subtypes (LumA and LumB) compared with HER2 and basal subtypes. *n* indicates the number of samples in each group. Molecular subtypes: LumA (luminal A: ER^+^/PR^+^/HER2^−^, low proliferation), LumB (luminal B: ER^+^ with high proliferation or HER2^+^), HER2 (HER2 enriched: ER^−^/PR^−^/HER2^+^), basal (basal-like: triple-negative, ER^−^/PR^−^/HER2^−^). **b** Correlation between ex4:3 bp expression and developmental lineage scores in TCGA. Ectoderm score shows the strongest positive correlation with ex4:3 bp expression, particularly in ER^+^ samples (*r* = 0.44) than in the ER^−^ samples (*r* = 0.32). By contrast, mesoderm and endoderm scores showed weaker or negative correlations, whereas the stemness score showed weak correlations in opposite directions for ER^+^ and ER^−^ samples. **c** Independent validation using CPTAC breast cancer data. Similar pattern of ex4:3 bp isoform distribution across molecular subtypes as observed in TCGA. **d** Analysis of MBC project data. Left: higher ex4:3 bp expression in ER^+^ samples than in ER^−^ samples (*P* = 4 × 10^−5^). Right: significantly lower ex4:3 bp expression in ER^+^ samples with distant lymph node metastasis (*P* = 4 × 10^−2^), suggesting that decreased expression of this isoform correlates with metastatic progression.

We next investigated the clinical relevance of *RPS24* ex4:3 bp isoform expression patterns in cohorts of patients with breast cancer. Analysis of TCGA data revealed significantly higher ex4:3 bp expression in luminal subtypes (LumA and LumB) than in other molecular subtypes (Fig. 5a). This observation aligns with our cell line findings, as luminal subtypes are characteristically ER+. The molecular subtype-specific patterns observed in TCGA were also independently validated in the CPTAC dataset (Fig. 5c).

We also examined correlations between ex4:3 bp expression and various developmental lineage scores in TCGA data, including ectoderm, mesoderm, endoderm and stemness scores (Fig. 5b). Among these, the ectoderm score showed a positive correlation with ex4:3 bp expression, particularly pronounced in ER^+^ samples (*r* = 0.44) compared with ER^−^ samples (*r* = 0.32). By contrast, mesoderm and endoderm scores showed weaker or negative correlations in both ER^+^ and ER^−^ samples. This specific association with ectoderm score suggests that the ex4:3 bp isoform may be linked to the maintenance of epithelial differentiation status in breast cancer, which is consistent with the epithelial origin of mammary tissue.

Considering that, typically, ER^+^ breast cancer has a more favorable prognosis than ER^−^ breast cancer does, we examined whether patient survival differed on the basis of the ex4:3 bp expression status using the TCGA data. However, no significant correlation was observed with overall survival (*P* = 0.1) or cancer stages (*P* = 0.7) (Supplementary Fig. 6). Interestingly, while the ex4:3 bp isoform did not show prognostic value in terms of overall survival or staging in the primary tumor setting, analysis of the MBC project data revealed some clinical associations of potential interest (Fig. 5d). The MBC data confirmed higher ex4:3 bp expression in ER^+^ samples compared with ER^−^ samples (*P* < 0.05) and showed an association between lower ex4:3 bp expression and distant lymph node metastasis status in ER^+^ samples (*P* < 0.05).

## Discussion

**Fig. 6:**
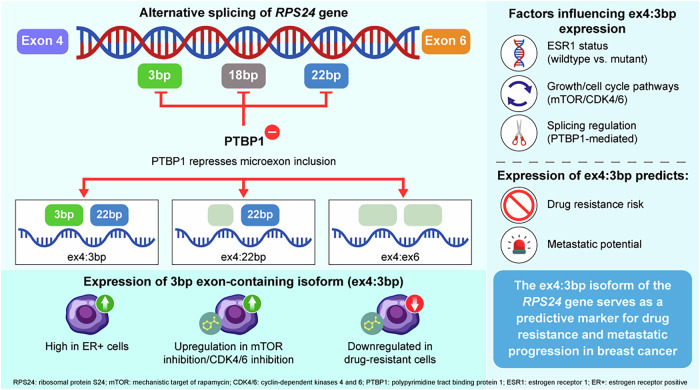
Integrated model of *RPS24* AS regulation and its clinical implications in breast cancer. The schematic summarizes regulatory mechanisms and clinical implications of the *RPS24* ex4:3 bp isoform in ER^+^ breast cancer. Inclusion of the 3-bp microexon (ex4:3 bp isoform) is repressed by PTBP1 binding to intronic regions, while ER signaling and cell cycle/growth pathway inhibition (via mTOR and CDK4/6 inhibitors) promote its expression. The ex4:3 bp isoform is downregulated in drug-resistant and metastatic cells but upregulated in response to targeted therapy, acting as a dynamic biomarker. High ex4:3 bp expression is associated with ER^+^ status, increased epithelial differentiation and sensitivity to treatment, while reduced expression correlates with drug resistance and metastatic potential. This isoform therefore serves as a predictive marker for therapeutic response and disease progression in breast cancer.

Our comprehensive investigation into *RPS24* AS in breast cancer reveals a complex regulatory network centered on the ex4:3 bp microexon isoform (Fig. 6). This study highlights three major findings: (1) the ex4:3 bp isoform serves as a molecular sensor for ER signaling and cell cycle regulation, (2) PTBP1 functions as a key upstream regulator controlling microexon inclusion and (3) the ex4:3 bp isoform exhibits potential as a predictive biomarker for drug resistance and epithelial differentiation status.

Our data demonstrate a strong dependency of the ex4:3 bp isoform on the *ESR1* gene, supported by multiple lines of evidence, including *ESR1* gene expression correlation, its elevated expression in cells where *ESR1* is essential for survival and the effects of *ESR1* gene mutations (Fig. 2). Building on recent reports that ER-alpha (encoded by *ESR1*) possesses post-transcriptional regulatory functions^25^, we discovered that ER-alpha interacts with RPS24 at multiple levels: through protein–protein interactions, RNA binding and its impact on cell fitness. This sophisticated interplay reveals a key regulatory mechanism where *RPS24* AS actively participates in maintaining ER^+^ breast cancer cell identity and survival, extending beyond merely being a downstream target of ER signaling.

Our systematic analysis identified PTBP1 as a critical repressor of *RPS24* microexon inclusion (Fig. 4). Computational predictions, CLIP-seq validation and functional studies collectively establish PTBP1 as a key regulatory node. PTBP1’s binding to polypyrimidine tracts upstream of all three microexons, coupled with its role in promoting exon skipping during cellular differentiation^27,28^, suggests *RPS24* splicing is integrated into broader developmental programs. Incomplete rescue after PTBP1 depletion indicates the involvement of additional regulatory factors, consistent with models of collaborative RNA-binding protein control over microexon splicing^29–31^. The *RPS24* microexons modify the C-terminal region, creating isoforms with distinct amino acid compositions. The negative correlation between ex4:3 bp transcript levels and RPS24 protein abundance suggests complex post-translational regulation. Upregulation of ex4:3 bp after mTOR and CDK4/6 inhibition highlights this C-terminal region as a signaling convergence point for growth control pathways. This parallels findings for *RPS26*, where the C-terminal domain integrates energy metabolism and AMPK–mTOR signaling^32^, indicating that ribosomal protein C termini may act as regulatory hubs linking *RPS24* AS to ER signaling, growth and cell cycle pathways.

Consistent downregulation of ex4:3 bp across diverse drug resistance models suggests its involvement in a common resistance pathway. Although ex4:3 bp isoform shows potential as a biomarker for monitoring treatment response, large-scale prospective clinical validation using an objective measuring method will be required for clinical implementation. For this, our high-resolution detection method would be helpful.

A correlation between ex4:3 bp expression and ectoderm scores (especially in ER^+^ samples) suggests a role in maintaining epithelial differentiation. Reduced ex4:3 bp expression being associated with metastatic progression in ER^+^ breast cancers further provides a mechanistic link to epithelial–mesenchymal transition. However, its lack of correlation with overall survival or primary tumor staging indicates that ex4:3 bp is a dynamic biomarker primarily predicting treatment response rather than universal prognosis.

In summary, our findings reveal *RPS24* AS as a sophisticated regulatory mechanism integrating multiple signaling pathways critical to breast cancer biology. The ex4:3 bp isoform acts as a molecular sensor responsive to ER signaling, cell cycle control and therapeutic interventions, regulated by both transcriptional (ER-alpha) and post-transcriptional (PTBP1) factors. C-terminal modifications from *RPS24* AS create functional diversity, with distinct isoforms correlating with protein abundance, supporting the emerging concept of ‘specialized ribosomes’ that fine-tune translation on the basis of cellular context.

Key limitations include the need for mechanistic details of ER-alpha-mediated *RPS24* splicing regulation, full characterization of the regulatory network beyond PTBP1 and functional consequences of different RPS24 protein isoforms. Future studies should focus on: (1) defining direct ER-alpha–*RPS24* RNA interactions, (2) identifying additional PTBP1-cooperating regulatory factors, (3) characterizing functional differences between RPS24 protein isoforms and (4) validating clinical utility in prospective trials.

## Supplementary information

Supplementary Information

## Data Availability

The data generated and analyzed during the current study are available from the corresponding author upon reasonable request.
