# Supplementary material for: RPS24 microexon isoform as a novel biomarker for estrogen receptor-positive breast cancer progression and therapeutic resistance
Source: Exp Mol Med. 2025 Nov 15;57(11):2608–18. doi: 10.1038/s12276-025-01578-y (PMC12686416; doi:10.1038/s12276-025-01578-y)
Supplement: Supplementary file 1 — Supplementary Information [file 12276_2025_1578_MOESM1_ESM.pdf]

Supplementary Figure 1. Visualization of *RPS24* splicing patterns in breast cancer cell lines from CCLE RNA-seq data. UCSC Genome Browser screenshot showing read coverage across the *RPS24* gene region containing the three microexons (3bp, 18bp, and 22bp) in 51 breast cancer cell lines. Each horizontal track represents a different cell line, with reads mapped to the microexon region displayed in various colors. The 3bp and 22bp exons show variable expression across cell lines, while the 18bp exon shows no detectable expression in any of the cell lines examined. This visual representation confirms the absence of 18bp exon inclusion in the *RPS24* transcripts of breast cancer cells. Cell lines are arranged in the same order as in Figure 1a, with arrows indicating the different junction types: green arrow for ex4:3bp junctions, cyan arrow for ex4:22bp junctions, and orange arrow for ex4:ex6 junctions.

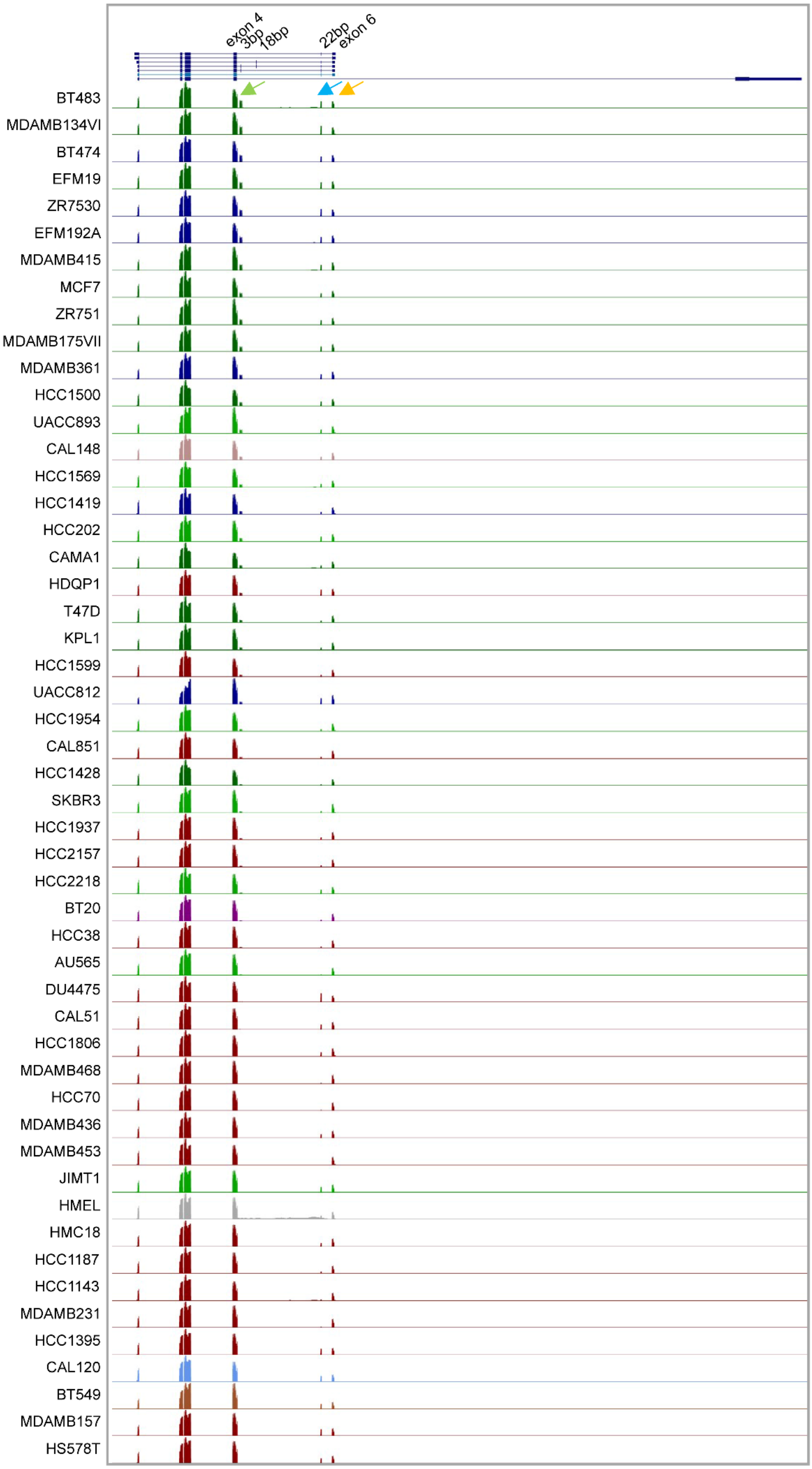
exon 4
3bp
18bp
22bp
exon 6
BT483
MDAMB134VI
BT474
EFM19
ZR7530
EFM192A
MDAMB415
MCF7
ZR751
MDAMB175VII
MDAMB361
HCC1500
UACC893
CAL148
HCC1569
HCC1419
HCC202
CAMA1
HDQP1
T47D
KPL1
HCC1599
UACC812
HCC1954
CAL851
HCC1428
SKBR3
HCC1937
HCC2157
HCC2218
BT20
HCC38
AU565
DU4475
CAL51
HCC1806
MDAMB468
HCC70
MDAMB436
MDAMB453
JIMT1
HMEL
HMC18
HCC1187
HCC1143
MDAMB231
HCC1395
CAL120
BT549
MDAMB157
HS578T

Supplementary Figure 2. Cross-validation of *RPS24* AS isoform measurements across multiple breast cancer RNA-seq datasets. Comparison between CCLE data (51 breast cancer cell lines) and four independent GEO datasets (GSE48213, GSE58135, GSE73526, and PRJEB30617). The numbers in parentheses indicate the number of overlapping cell lines between CCLE and each GEO dataset out of the total number of cell lines in each GEO dataset. The table shows Pearson correlation coefficients (r) for each *RPS24* AS isoform (ex4:3bp, ex4:22bp, and ex4:ex6) between CCLE and the four datasets. The ex4:3bp isoform shows the strongest correlation across all datasets (median r=0.92), while ex4:22bp and ex4:ex6 also demonstrate strong concordance (median r=0.81 for both). These high correlations validate the reliability of our *RPS24* AS isoform quantification method across different sequencing platforms and experimental conditions.

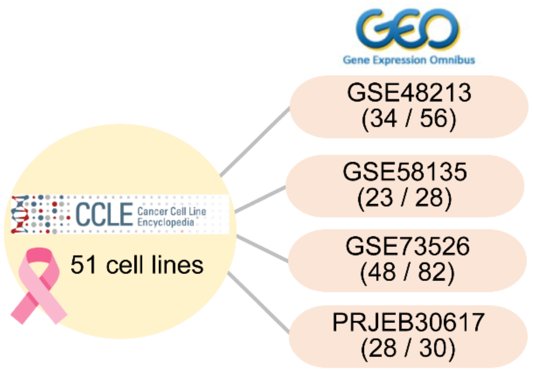

r	ex4:3bp	ex4:22bp	ex4:ex6
GSSE48213	0.86	0.64	0.75
GSE58135	0.93	0.81	0.82
GSE73526	0.94	0.84	0.86
PRJEB30617	0.92	0.80	0.80
**median**	**0.92**	**0.81**	**0.81**

Supplementary Figure 3. Correlation between *RPS24* AS isoform proportions and RPS24 gene expression at RNA and protein levels. Scatter plots show the relationship between each *RPS24* AS isoform (ex4:3bp in green, ex4:22bp in cyan, and ex4:ex6 in orange) and *RPS24* gene expression in both CCLE breast cancer cell lines (n=30, left panels) and CPTAC breast cancer patient samples (n=107, right panels). Each data point represents a sample, with Pearson correlation coefficients (r) indicated in the top left corner of each plot. The ex4:3bp isoform shows no significant correlation with *RPS24* mRNA levels (r=0.10 in CCLE, r=0.10 in CPTAC) but exhibits negative correlations with protein levels (r=-0.38 in CCLE, r=-0.51 in CPTAC). The ex4:22bp isoform shows weak correlations with both mRNA and protein levels. In contrast, the ex4:ex6 isoform demonstrates varying correlations across datasets, with a notable positive correlation with protein levels in CPTAC data (r=0.55).

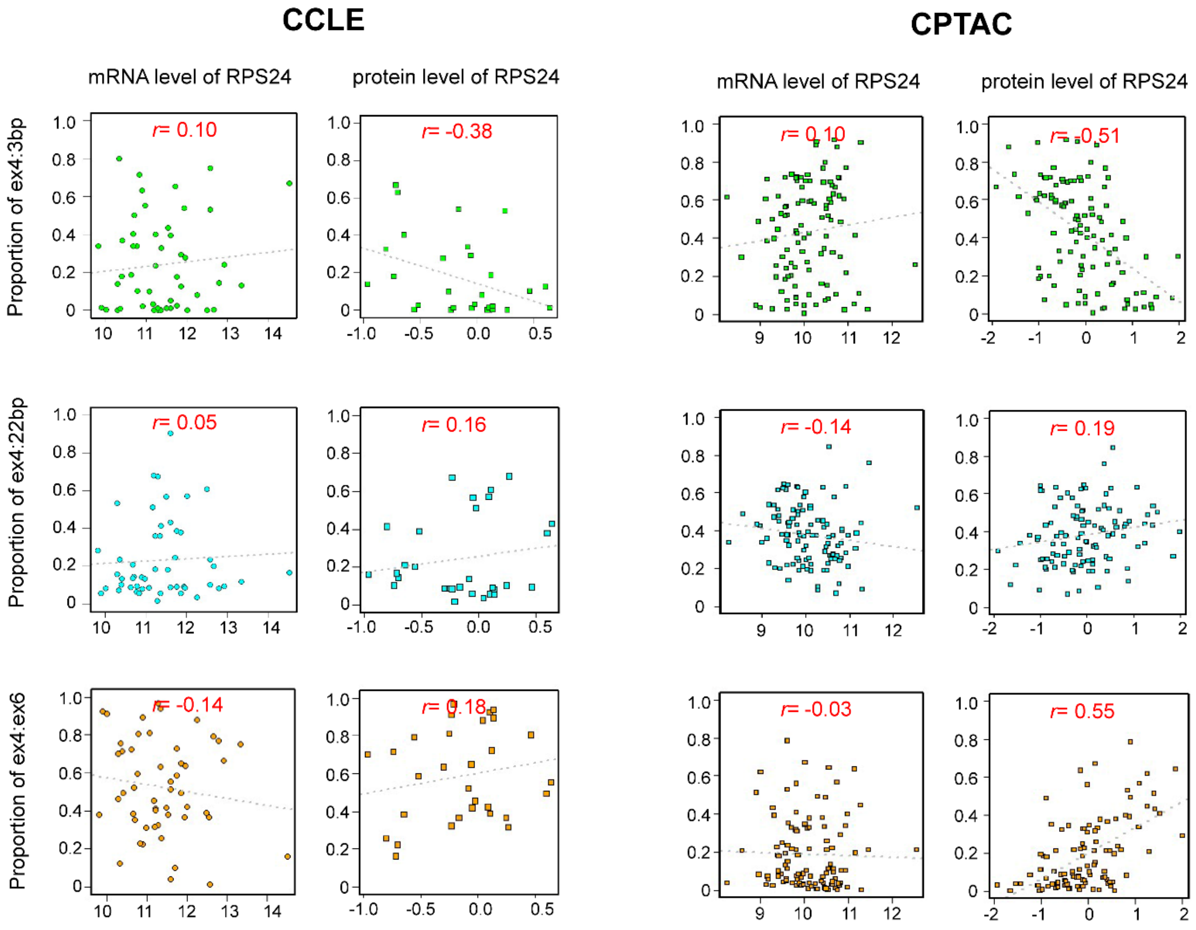

Supplementary Figure 4. Distribution of *RPS24* AS isoforms across breast cancer molecular subtypes. Box plots represent the proportions of three *RPS24* AS isoforms (ex4:3bp, ex4:22bp, and ex4:ex6) across different breast cancer molecular subtypes. The subtypes include luminal, HER2-amplified (HER2_amp), luminal HER2-amplified (luminal_HER2_amp), and two basal subtypes (basal_A and basal_B). The y-axis represents the proportion of each isoform, ranging from 0 to 1. The p-values at the top of each panel indicate the statistical significance of the differences observed across subtypes, as determined by ANOVA.

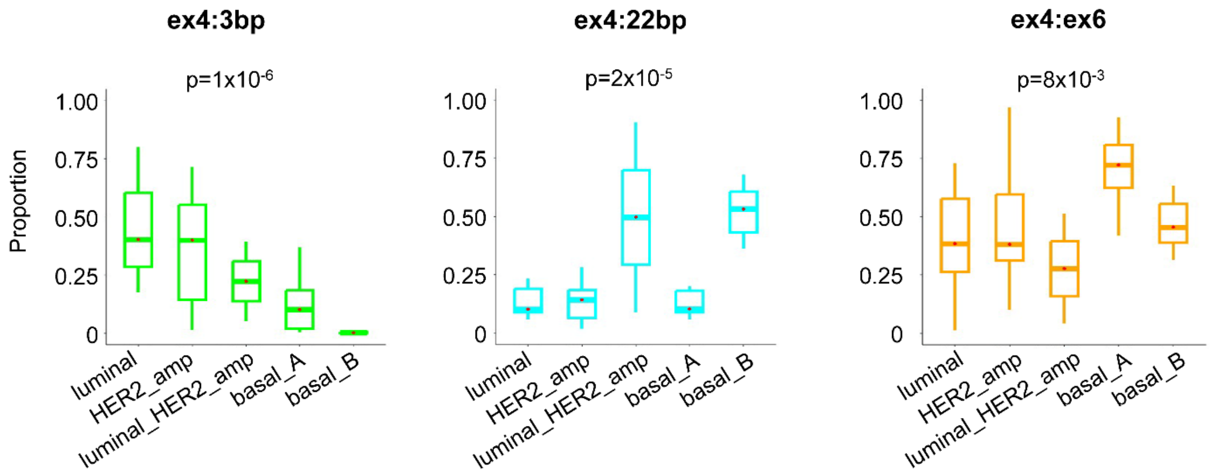

Supplementary Figure 5. Strong Association Between *RPS24* ex4:3bp Isoform and *ESR1* Gene Status in Breast Cancer. (a) Plots demonstrating that *ESR1* is among the top genes associated with the ex4:3bp isoform. Left: Genes sorted by mRNA expression correlation, with *ESR1* showing strong positive correlation (red dot). Right: Genes sorted by gene dependency score (RNAi), with *ESR1* showing strong negative correlation (red dot), indicating cells with high ex4:3bp expression are more dependent on *ESR1*. **(b-d)** Impact of *ESR1* alterations on *RPS24* ex4:3bp isoform expression across multiple datasets: **(b)** GSE89888: Proportion of ex4:3bp isoform in T47D and MCF7 cells with WT or mutant *ESR1* (Y537S, D538G), with or without estrogen treatment, showing significant reduction in ex4:3bp in mutant cells. **(c)** GSE206185: Similar analysis in MCF7 cells, confirming reduced ex4:3bp expression with *ESR1* mutations regardless of estrogen treatment. **(d)** GSE266408: Analysis in T47D cells expressing various *ESR1* variants, including WT, truncated *ESR1* (ΔCTD), and *ESR1* fusion proteins (DAB2, LPP, SOX9, YAP1), all showing significantly reduced ex4:3bp expression compared to WT, with or without fulvestrant treatment. P-values are denoted as ***, **, and * when ≤ 0.0005, ≤ 0.005, and ≤ 0.05, respectively.

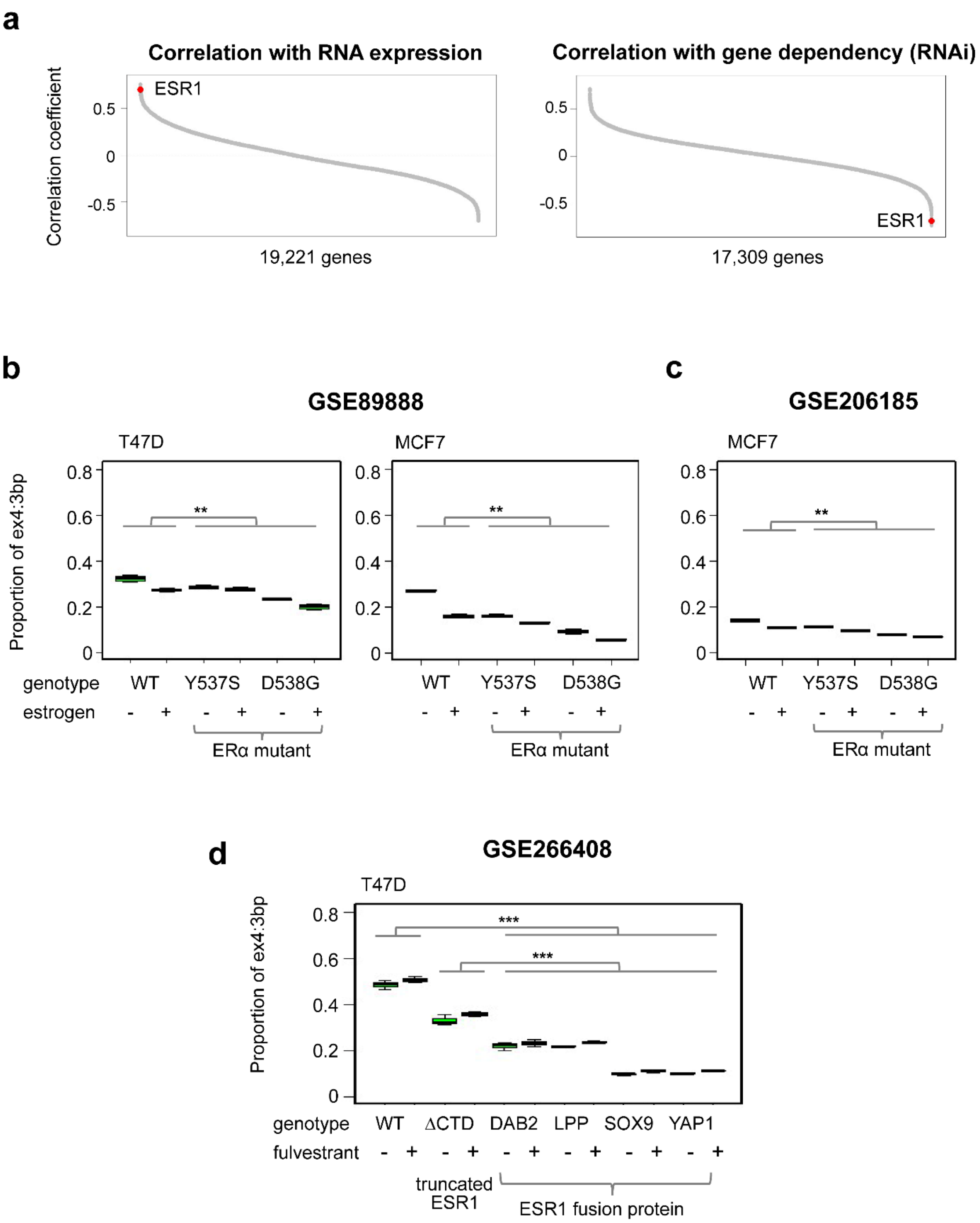
a
Correlation with RNA expression
Correlation with gene dependency (RNAi)
Correlation coefficient
0.5
0
-0.5
ESR1
19,221 genes
17,309 genes
b
GSE89888
T47D
MCF7
Proportion of ex4:3bp
0.8
0.6
0.4
0.2
0
**
genotype
WT
Y537S
D538G
estrogen
- + - + - +
ERα mutant
c
GSE206185
MCF7
d
GSE266408
T47D
***
genotype
WT
ΔCTD
DAB2
LPP
SOX9
YAP1
fulvestrant
- + - + - + - + - + - +
truncated ESR1
ESR1 fusion protein

Supplementary Figure 6. Clinical associations of ex4:3bp isoform expression in TCGA breast cancer cohort. Left panel: Violin plots showing ex4:3bp isoform proportions between estrogen receptor-positive (ER+) and estrogen receptor-negative (ER-) samples. ER+ samples exhibit significantly higher ex4:3bp expression compared to ER- samples ($p=7\times10^{-89}$). Middle panel: Box plots displaying ex4:3bp isoform proportions across cancer stages (stages 1-5). No significant association was observed between ex4:3bp expression and cancer staging (p=0.70). Right panel: Kaplan-Meier survival curves comparing overall survival between patients with high (red) and low (yellow) ex4:3bp expression levels. No significant difference in overall survival was observed between the two groups (p=0.10). Statistical significance was determined using appropriate tests for each analysis type.

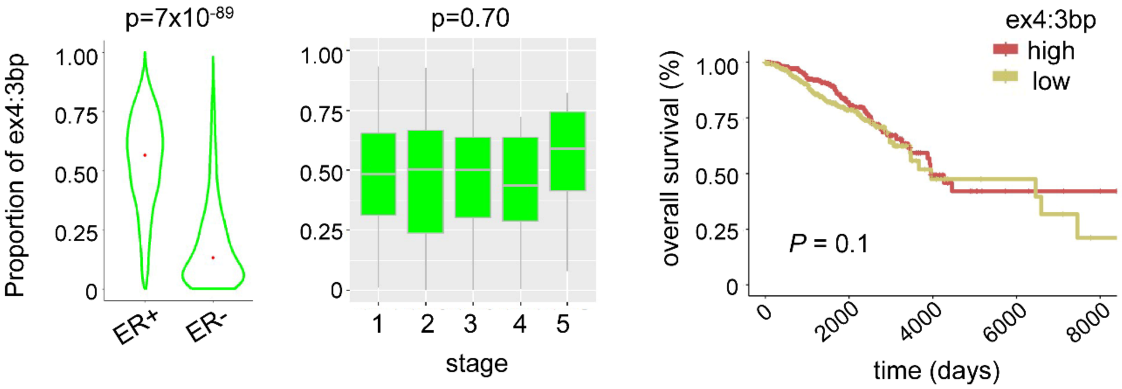

Supplementary Table 1. Proportions of each *RPS24* AS isoform upon knockdown of the respective splicing factor in HeLa cells. This table presents the relative abundance of each *RPS24* splicing isoform (ex4:3bp, ex4:22bp, ex4:ex6, and other) in HeLa cells following siRNA-mediated knockdown of individual splicing factors. Isoform quantification was based on analysis of splice junction usage from publicly available RNA-seq data (E-MTAB-11202). Values represent the proportion of each isoform relative to total *RPS24* AS events. Genes are sorted in descending order based on the expression of the ex4:22bp isoform. “Other” includes all minor junction combinations not classified as the three major isoforms.

target gene for knockdown	ex4:3bp	**ex4:22bp**	ex4:ex6	other
U2AF1	0.062	0.729	0.197	0.013
PTBP1	0.046	0.605	0.347	0.002
PPM1G	0.049	0.595	0.356	0.000
HNRNPU	0.023	0.526	0.449	0.001
SRSF3	0.020	0.525	0.454	0.001
KAT2B	0.010	0.503	0.486	0.001
RBM39	0.018	0.500	0.480	0.002
MOV10	0.034	0.495	0.471	0.000
THOC6	0.010	0.485	0.504	0.000
EP300	0.018	0.480	0.502	0.000
RBM25	0.014	0.467	0.518	0.001
HNRNPA2B1	0.020	0.460	0.519	0.001
DHX15	0.005	0.443	0.550	0.001
AA7	0.028	0.439	0.532	0.000
CTCF	0.034	0.432	0.533	0.002
ADARB1	0.012	0.429	0.559	0.000
RPS6KA5	0.034	0.413	0.553	0.001
DHX8	0.014	0.413	0.570	0.004
DDX3X	0.009	0.411	0.580	0.001
SNRNP35	0.012	0.405	0.582	0.002
KAT2A	0.016	0.387	0.597	0.000
U2SURP	0.009	0.381	0.609	0.001
PPIH	0.061	0.373	0.565	0.001
RBM17	0.010	0.369	0.620	0.001
BRD4	0.015	0.368	0.617	0.000
BRINP1	0.004	0.365	0.631	0.000
PRPF40A	0.007	0.360	0.633	0.001
HDAC3	0.013	0.359	0.627	0.001
SETD2	0.018	0.353	0.628	0.001
KDM1A	0.009	0.351	0.640	0.000
HDAC4	0.030	0.348	0.621	0.000
YBX1	0.010	0.343	0.647	0.000
BMI1	0.013	0.342	0.644	0.000
DHX35	0.015	0.339	0.646	0.000
PRMT1	0.007	0.334	0.658	0.001
EED	0.024	0.330	0.645	0.001
CLK4	0.024	0.321	0.654	0.001
DDX17	0.008	0.316	0.675	0.000
ALYREF	0.012	0.313	0.674	0.000
BCAS2	0.009	0.312	0.677	0.001
CHERP	0.021	0.312	0.665	0.002
MECP2	0.021	0.309	0.668	0.002

PDCD7	0.007	0.309	0.684	0.000
FUS	0.016	0.305	0.679	0.000
HNRNPH3	0.006	0.305	0.688	0.001
SNRNP48	0.018	0.302	0.679	0.001
HSPA8	0.016	0.299	0.684	0.000
RALY	0.012	0.297	0.691	0.000
PABPN1	0.009	0.295	0.696	0.001
ZNF326	0.009	0.293	0.697	0.001
KAT5	0.013	0.291	0.696	0.000
DEK	0.003	0.289	0.707	0.000
EXOSC4	0.015	0.289	0.696	0.001
DDX10	0.007	0.287	0.707	0.000
SRPK1	0.032	0.286	0.682	0.000
ZRSR1	0.005	0.285	0.708	0.001
TRA2B	0.009	0.284	0.705	0.001
TAF15	0.005	0.282	0.712	0.000
DDX5	0.017	0.281	0.701	0.001
CBX3	0.016	0.281	0.702	0.001
FAM50A	0.004	0.281	0.715	0.001
CTNNBL1	0.020	0.280	0.700	0.001
DHX33	0.008	0.278	0.714	0.000
FRG1	0.005	0.277	0.718	0.000
SKIV2L2	0.008	0.275	0.716	0.001
HNRNPM	0.010	0.275	0.714	0.000
PPIG	0.007	0.271	0.722	0.000
CPSF6	0.021	0.269	0.708	0.002
IGF2BP3	0.009	0.269	0.721	0.001
NUDT21	0.010	0.269	0.721	0.001
RECQL5	0.022	0.269	0.709	0.001
METTL14	0.011	0.268	0.721	0.001
HNRNPA1	0.025	0.266	0.709	0.000
U2AF2	0.013	0.263	0.722	0.003
PRMT5	0.017	0.261	0.722	0.001
HNRNPUL1	0.009	0.260	0.730	0.001
CHD1	0.017	0.260	0.722	0.001
DIS3	0.011	0.254	0.734	0.001
HSPA1A	0.007	0.254	0.738	0.001
DHX9	0.010	0.253	0.737	0.000
SPEN	0.022	0.250	0.727	0.001
MATR3	0.015	0.248	0.736	0.001
DDX46	0.012	0.248	0.740	0.000
DNMT1	0.009	0.246	0.744	0.001
SETD1A	0.006	0.246	0.748	0.000

HNRNPH1	0.013	0.244	0.742	0.001
PQBP1	0.004	0.244	0.752	0.000
DHX30	0.016	0.243	0.741	0.000
PABPC1	0.008	0.242	0.750	0.000
HNRNPA0	0.008	0.241	0.750	0.000
RBM15	0.010	0.241	0.748	0.000
HFM1	0.016	0.241	0.742	0.001
SYNCRIP	0.008	0.240	0.751	0.001
WTAP	0.004	0.240	0.756	0.001
ISY1	0.021	0.239	0.739	0.001
NCBP2	0.006	0.239	0.755	0.000
ZCRB1	0.014	0.236	0.748	0.002
WBP11	0.010	0.236	0.754	0.000
HMGA1	0.010	0.235	0.754	0.000
PPIE	0.010	0.235	0.755	0.001
IK_r1	0.007	0.235	0.757	0.001
RBM8A	0.003	0.234	0.762	0.001
PPWD1	0.012	0.234	0.754	0.000
IK_r2	0.011	0.234	0.755	0.000
SNRNP40	0.013	0.232	0.755	0.000
DDX11	0.011	0.231	0.757	0.001
ILF3	0.039	0.231	0.729	0.000
SRSF2	0.013	0.230	0.756	0.001
EWSR1	0.011	0.229	0.758	0.001
EHMT2	0.015	0.229	0.754	0.001
ELAVL1	0.014	0.229	0.756	0.001
ASCL1	0.012	0.225	0.762	0.000
THOC2	0.006	0.225	0.769	0.000
ZNF207	0.004	0.225	0.770	0.001
TIA1	0.014	0.224	0.761	0.000
SYF2	0.014	0.223	0.762	0.001
LPAR1	0.019	0.222	0.759	0.000
CDK12	0.017	0.222	0.760	0.001
KHDRBS1	0.010	0.222	0.768	0.000
DDX52	0.013	0.220	0.767	0.000
SRSF4	0.009	0.220	0.771	0.000
TCERG1	0.003	0.218	0.777	0.002
THOC1	0.010	0.217	0.772	0.000
THOC5	0.005	0.217	0.778	0.000
SRSF7	0.020	0.217	0.764	0.000
HSPA5	0.010	0.217	0.772	0.001
TCERG1L	0.004	0.214	0.781	0.000
ASH2L	0.005	0.214	0.780	0.001

CCDC12	0.006	0.213	0.781	0.000
HNRNPF	0.013	0.213	0.773	0.001
PLRG1	0.009	0.213	0.778	0.000
AA5	0.006	0.212	0.782	0.000
MAGOH	0.006	0.211	0.782	0.001
ZRSR2	0.003	0.211	0.786	0.000
RBM6	0.009	0.210	0.781	0.000
DDX28	0.010	0.210	0.780	0.001
THOC3	0.004	0.210	0.786	0.000
ACIN1	0.023	0.209	0.768	0.000
SNRNP70	0.013	0.208	0.777	0.001
BUD31	0.007	0.206	0.787	0.000
CPSF2	0.016	0.206	0.778	0.000
LSM7	0.007	0.205	0.787	0.000
SRSF9	0.004	0.205	0.790	0.001
TDRD9	0.008	0.205	0.786	0.001
SRRM1	0.005	0.203	0.791	0.001
DICER1	0.012	0.203	0.785	0.000
PHC1	0.012	0.203	0.784	0.001
SF3B6	0.004	0.203	0.792	0.001
HNRNPA3	0.011	0.203	0.785	0.001
RNPC3	0.013	0.202	0.785	0.000
MORF4L1	0.006	0.201	0.792	0.000
RBMX2	0.005	0.200	0.794	0.001
CARM1	0.006	0.200	0.794	0.001
SRRM3	0.007	0.199	0.794	0.000
DDX41	0.012	0.199	0.789	0.000
BUD13	0.004	0.199	0.796	0.001
NAB2	0.001	0.199	0.800	0.000
CACTIN	0.018	0.198	0.784	0.000
SNIP1	0.013	0.197	0.789	0.000
HDAC2	0.009	0.197	0.793	0.001
CWC27	0.020	0.197	0.783	0.001
SF3B2	0.002	0.197	0.801	0.000
SRSF6	0.008	0.196	0.794	0.002
WBP4	0.026	0.196	0.778	0.001
PRPF4	0.009	0.196	0.795	0.000
SLU7	0.007	0.195	0.795	0.003
EZH2	0.007	0.194	0.798	0.001
CDC40	0.011	0.194	0.794	0.001
DHX16	0.009	0.194	0.797	0.000
MBD2	0.004	0.193	0.803	0.001
HNRNPH2	0.009	0.193	0.797	0.000

DDX24	0.014	0.192	0.793	0.001
HNRNPD	0.011	0.192	0.796	0.000
RBM5	0.012	0.192	0.795	0.001
DDX39B	0.008	0.192	0.799	0.000
PRPF31	0.006	0.191	0.802	0.000
PRPF39	0.006	0.191	0.802	0.000
YBX3	0.006	0.191	0.801	0.001
SRRM2	0.011	0.191	0.797	0.001
BAZ1B	0.013	0.191	0.796	0.000
HNRNPDL	0.008	0.191	0.800	0.001
SF1	0.003	0.191	0.805	0.002
EIF4A3	0.007	0.190	0.803	0.000
CXorf56	0.007	0.190	0.802	0.001
SNRPB2	0.025	0.189	0.786	0.000
SNRNP25	0.006	0.188	0.806	0.000
SIRT1	0.007	0.188	0.805	0.000
LUC7L3	0.005	0.188	0.807	0.000
CRNKL1	0.007	0.188	0.804	0.001
SNRNP27	0.007	0.188	0.805	0.000
THRAP3	0.009	0.188	0.803	0.000
SRSF5	0.015	0.187	0.797	0.000
DDX12P	0.007	0.187	0.805	0.001
MFAP1	0.005	0.186	0.809	0.000
C9orf78	0.005	0.186	0.810	0.000
NOSIP	0.006	0.185	0.809	0.001
AQR	0.001	0.184	0.814	0.001
FAM32A	0.007	0.183	0.809	0.001
PPIL2	0.006	0.183	0.810	0.001
PPIL3	0.006	0.182	0.811	0.000
LENG1	0.007	0.182	0.811	0.001
AA6	0.001	0.179	0.819	0.001
CLK1	0.015	0.179	0.806	0.000
PRPF19	0.003	0.179	0.818	0.000
NCBP1	0.017	0.177	0.805	0.001
FAM50B	0.009	0.177	0.813	0.001
CDK10	0.014	0.177	0.808	0.000
CDC5L	0.007	0.177	0.816	0.000
AA8	0.007	0.176	0.816	0.001
HDAC1	0.012	0.176	0.812	0.000
CWC22_r1	0.006	0.175	0.819	0.000
SRPK2_r2	0.025	0.175	0.800	0.000
SART1	0.011	0.174	0.814	0.001
FUBP3	0.007	0.173	0.818	0.001

PHC2	0.007	0.173	0.820	0.000
AA4	0.002	0.173	0.826	0.000
XAB2_r1	0.008	0.172	0.818	0.002
RNPS1	0.012	0.172	0.816	0.000
SNRPC	0.009	0.172	0.819	0.000
HNRNPC	0.014	0.171	0.815	0.000
LSM6	0.004	0.171	0.825	0.000
PRPF18	0.010	0.170	0.819	0.001
CIRBP	0.005	0.170	0.825	0.000
SRSF1	0.011	0.169	0.820	0.000
PRPF4B	0.008	0.169	0.823	0.000
AGO2	0.008	0.169	0.822	0.001
KIN	0.010	0.168	0.821	0.001
HNRNPLL	0.005	0.168	0.826	0.000
SMARCA4	0.014	0.168	0.818	0.000
HNRNPUL2	0.006	0.168	0.826	0.001
NSRP1	0.005	0.167	0.829	0.000
HNRNPR	0.010	0.165	0.824	0.000
TET1	0.007	0.163	0.828	0.001
CWC15	0.003	0.163	0.833	0.001
CWC22_r2	0.004	0.161	0.835	0.000
KDM4B	0.002	0.161	0.837	0.000
SRPK2_r1	0.020	0.159	0.820	0.001
CLK3	0.008	0.159	0.833	0.000
DHX38	0.002	0.159	0.839	0.001
AA3	0.002	0.158	0.839	0.000
SNU13	0.009	0.158	0.833	0.000
DDX23	0.006	0.158	0.835	0.002
C19orf43	0.001	0.157	0.842	0.000
FTO	0.004	0.156	0.840	0.000
NONO	0.004	0.154	0.840	0.001
ESRP2	0.004	0.154	0.843	0.000
SRSF11	0.007	0.154	0.839	0.000
DDX31	0.004	0.153	0.842	0.001
METTL3	0.006	0.152	0.842	0.001
DIS3L	0.014	0.151	0.834	0.000
BUB3	0.011	0.149	0.840	0.000
RBM10	0.003	0.149	0.848	0.000
DGCR14	0.008	0.148	0.843	0.000
USP39	0.006	0.148	0.841	0.004
XAB2_r2	0.007	0.146	0.844	0.003
LSM3	0.005	0.143	0.851	0.001
CLK2	0.011	0.143	0.846	0.000

SNW1	0.002	0.142	0.856	0.000
RBM22	0.005	0.142	0.852	0.001
ZMAT5	0.002	0.141	0.856	0.000
YTHDC1	0.003	0.140	0.858	0.000
DBR1	0.001	0.139	0.859	0.001
SF3A3	0.004	0.132	0.864	0.001
WDR83	0.005	0.132	0.863	0.000
HNRNPAB	0.003	0.130	0.867	0.000
HNRNPK	0.010	0.130	0.859	0.000
SNRNP200	0.020	0.130	0.850	0.000
HDAC6	0.007	0.128	0.865	0.000
TFIP11	0.004	0.126	0.869	0.000
ZNF830	0.003	0.125	0.871	0.000
HNRNPCL1	0.005	0.125	0.869	0.000
AA9	0.006	0.124	0.869	0.000
DHX57	0.000	0.124	0.875	0.002
LSM2	0.012	0.123	0.864	0.000
SF3A2	0.003	0.123	0.874	0.000
DNAJC8	0.007	0.122	0.871	0.000
SNRPA	0.004	0.121	0.875	0.000
PRPF8_r2	0.007	0.121	0.872	0.000
SFPQ	0.003	0.121	0.875	0.001
CFAP20	0.007	0.119	0.875	0.000
CD2BP2	0.024	0.118	0.857	0.000
HNRNPL	0.004	0.117	0.879	0.000
CCAR1	0.002	0.117	0.880	0.001
DHX32	0.005	0.116	0.878	0.001
PRPF6	0.005	0.115	0.880	0.000
TXNL4A	0.002	0.114	0.884	0.000
SNRPE	0.002	0.113	0.884	0.001
PUF60	0.003	0.112	0.885	0.000
THOC7	0.013	0.111	0.876	0.001
SMU1_r2	0.003	0.110	0.886	0.000
ESRP1	0.005	0.109	0.886	0.000
ADAR	0.004	0.107	0.889	0.000
HTATSF1	0.006	0.103	0.891	0.001
SF3A1	0.004	0.101	0.895	0.000
TIAL1	0.003	0.100	0.897	0.000
PRPF8_r1	0.003	0.098	0.899	0.000
SNRPA1	0.002	0.098	0.900	0.001
ALKBH5	0.000	0.094	0.906	0.000
PRPF3	0.002	0.088	0.909	0.000
ILF2	0.002	0.087	0.911	0.000

SUV39H1	0.030	0.086	0.884	0.000
SMARCA2	0.002	0.085	0.913	0.000
SF3B4	0.003	0.085	0.912	0.000
LSM4	0.007	0.084	0.908	0.001
KIAA1429	0.003	0.084	0.913	0.000
SMNDC1	0.002	0.084	0.913	0.000
RBFOX2	0.020	0.081	0.899	0.000
PAXBP1	0.013	0.080	0.907	0.000
SNRPF	0.004	0.077	0.919	0.001
SNRPG	0.008	0.073	0.919	0.000
SF3B3	0.002	0.070	0.928	0.000
SMU1_r1	0.000	0.069	0.931	0.000
EFTUD2	0.001	0.065	0.934	0.000
SNRPD1	0.004	0.064	0.932	0.000
SNRPD2	0.006	0.062	0.932	0.000
SNRPB	0.003	0.049	0.947	0.001
SNRPD3	0.002	0.047	0.951	0.000
SF3B1_r1	0.002	0.046	0.952	0.000
SF3B1_r2	0.001	0.034	0.965	0.000
